# Time-Dependent Impact of Irreversible Electroporation on Pancreas, Liver, Blood Vessels and Nerves: A Systematic Review of Experimental Studies

**DOI:** 10.1371/journal.pone.0166987

**Published:** 2016-11-21

**Authors:** J. A. Vogel, E van Veldhuisen, P. Agnass, J. Crezee, F. Dijk, J. Verheij, T. M. van Gulik, M. R. Meijerink, L. G. Vroomen, K. P. van Lienden, M. G. Besselink

**Affiliations:** 1 Department of Surgery, Academic Medical Center, Amsterdam, the Netherlands; 2 Department of Radiation Therapy, Academic Medical Center, Amsterdam, the Netherlands; 3 Department of Pathology, Academic Medical Center, Amsterdam, the Netherlands; 4 Department of Experimental Surgery, Academic Medical Center, Amsterdam, the Netherlands; 5 Department of Radiology and Nuclear Medicine, VU University Medical Center, Amsterdam, the Netherlands; 6 Department of Radiology, Academic Medical Center, Amsterdam, the Netherlands; University of Szeged, HUNGARY

## Abstract

**Introduction:**

Irreversible electroporation (IRE) is a novel ablation technique in the treatment of unresectable cancer. The non-thermal mechanism is thought to cause mostly apoptosis compared to necrosis in thermal techniques. Both in experimental and clinical studies, a waiting time between ablation and tissue or imaging analysis to allow for cell death through apoptosis, is often reported. However, the dynamics of the IRE effect over time remain unknown. Therefore, this study aims to summarize these effects in relation to the time between treatment and evaluation.

**Methods:**

A systematic search was performed in Pubmed, Embase and the Cochrane Library for original articles using IRE on pancreas, liver or surrounding structures in animal or human studies. Data on pathology and time between IRE and evaluation were extracted.

**Results:**

Of 2602 screened studies, 36 could be included, regarding IRE in liver (n = 24), pancreas (n = 4), blood vessels (n = 4) and nerves (n = 4) in over 440 animals (pig, rat, goat and rabbit). No eligible human studies were found. In liver and pancreas, the first signs of apoptosis and haemorrhage were observed 1–2 hours after treatment, and remained visible until 24 hours in liver and 7 days in pancreas after which the damaged tissue was replaced by fibrosis. In solitary blood vessels, the tunica media, intima and lumen remained unchanged for 24 hours. After 7 days, inflammation, fibrosis and loss of smooth muscle cells were demonstrated, which persisted until 35 days. In nerves, the median time until demonstrable histological changes was 7 days.

**Conclusions:**

Tissue damage after IRE is a dynamic process with remarkable time differences between tissues in animals. Whereas pancreas and liver showed the first damages after 1–2 hours, this took 24 hours in blood vessels and 7 days in nerves.

## Introduction

Irreversible Electroporation (IRE) is a novel ablative technique for the treatment of unresectable soft tissue cancers. Ultra-short, high voltage pulses are applied through multiple electrodes, placed in and around the tumor to distort homeostasis in the targeted tissue through permeabilization of the cells’ membranes[1, 2]. Cell death is caused by this permeabilization, thermal damage or a combination of both[3, 4]. Contrary to thermal ablation techniques, IRE leaves the surrounding tissue intact, causing less collateral damage and thus leading to fewer side effects, less morbidity and a faster recovery[5]. Also, due to the non-thermal electroporation effect, treatment with IRE is not impaired by a heat-sink effect[6] where relatively cool blood flowing in large vessels through the ablation zone prohibits the attainment of effective temperatures around these vessels, leaving viable tumor tissue in situ [7]. Consequently, IRE can be beneficial in the treatment of unresectable tumors or tumors that are not eligible for thermal ablation therapies. Currently, the technique is extensively investigated in pancreatic, liver and urologic tumors and tissues[1, 5].

However, consensus on the mechanism of work of IRE has not been realized yet. As a result, contemporary literature is hesitant on defining this matter. Whether the effects of IRE are caused by a thermal or non-thermal mechanism remains unclear, since histological evaluation of tissue treated with IRE demonstrates characteristics of both necrosis and apoptosis, representing these two mechanisms of cell death[3, 6, 8–19]. Cell apoptosis is associated with tissue regeneration and less inflammatory response and therefore the most preferable effect. Yet, it may be unavoidable that IRE causes collateral, coagulative necrosis to some extent[3]. This can be explained by the secondary heating effect of IRE, whereas part of the electric energy is converted to thermal energy while passing through resistive tissue[4]. Furthermore, the influence of different settings (e.g. electric field strength, pulse number and duration) on pathological outcomes needs further examination. Several animal studies have aimed to clarify the mechanisms of IRE using a wide range of settings, and by comparing the effects of IRE with alternative techniques[20–23]. The main limitation of these studies is that specimen evaluation was performed at varying times after IRE-ablation. This may be problematic since cell death is a dynamic process by definition(23), and the question to which extent the time of histological evaluation determines the outcomes remains unanswered.

Therefore, the aim of this systematic review is to create an overview of the time-dependent effects of IRE, focusing on liver and pancreas, but also on nerves and blood vessels due to their close anatomical relation.

## Methods

This systematic review was performed according to the Preferred Reporting Items for Systematic Reviews[24] and Meta (PRISMA)-analyses guidelines[25].

### Study selection

**Fig 1:**
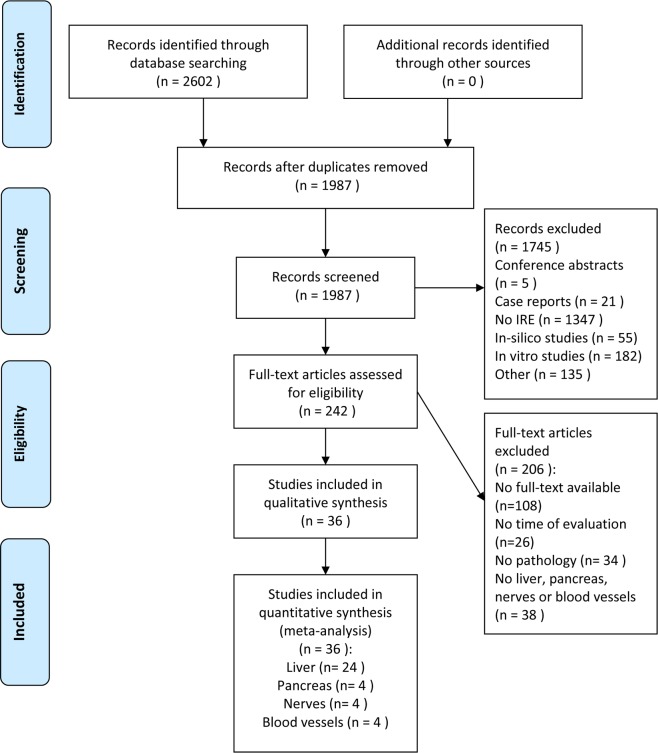
Flow diagram of study selection.

A systematic search was performed in Pubmed, Embase and the Cochrane Library for studies published in English language, from inception to October 1^st^, 2015. Different domains of medical subject headings (MeSH) terms and keywords were combined with ‘AND’, and within domains, the terms were combined with ‘OR’. The first domain included terms related to irreversible electroporation, whereas the second domain contained terms related to pathology. Terms were restricted to MeSH, title, abstract and keywords (a full description of the search strategy is available in S1 Appendix). Duplicates were removed and articles were screened by title, abstract and full-text for eligibility (Fig 1) based on predefined inclusion and exclusion criteria (S1 Table) by two authors independently (EV, JVo). Discordant judgements were addressed by discussion and consensus.

### Eligibility criteria

Included were studies concerning treatment with IRE in liver, pancreas, blood vessels or nerves of laboratory animals or patients reporting on macroscopic and/or histological findings and reporting on the time between IRE and tissue evaluation. Studies were required to have a description of treatment settings used. Excluded were in silico studies, articles considering reversible electroporation or considering electroporation as part of a multimodality treatment such as electrochemotherapy, studies describing IRE in pathological tissue and conference abstracts merely published in abstract form.

### Assessment of methodological quality

All of the selected studies were critically appraised for methodology according to the Office of Health Assessment and Translation risk of bias tool for human and animal studies (EV, JVo) [26]. Results of the critical appraisal are shown in S2 Table: Assessment of methodological quality of included studies.

### Data extraction

Data on study characteristics, design, type of animals (or human tissue), type of organ, sample size, IRE treatment settings, pathological findings and time between treatment and pathology evaluation were extracted (EV, JVo).

### Statistical analysis

A subspecies analysis was performed by considering animal species as subgroups in order to evaluate the differences in histopathological response in time between animal types (S3 Table). Due to the qualitative aspect of the study results, meta-analysis could not be performed for gross pathological and histopathological outcomes. Meta-analysis of the systemic effects of IRE was not possible due to a limited availability of data on these outcomes.

## Results

After removal of duplicates, screening by title, abstract and full text (Fig 1), 36 individual articles were included from 2602 search results. Included studies reported on IRE in liver (n = 24), pancreas (n = 4), blood vessels (n = 4) and nerves (n = 4) and involved over 440 animals (pigs, rats, goats and rabbits). No studies in humans were included. Results are presented per organ.

### Liver

The effects of IRE on liver tissue were investigated in 24 studies performing IRE *in vivo* using more than 260 animals[3, 6, 8–19, 27–36]. In 3 of these studies the exact number of animals tested was unclear[9, 10, 30]. Ablations were performed in pigs[3, 6, 8–12, 16–19, 27–29, 31, 32, 35], rats[13–15, 30, 36], goats[33] and rabbits[34]. All procedures were performed using general anaesthesia with adequate muscle relaxation. No animal died during treatment. One animal (pig) was excluded from the original analysis due to improper placement of the electrodes[8].

The IRE settings varied between 1 to 360 pulses, 360–3000 V/cm, and pulse length between 20 and 100 μs. Time between IRE and euthanasia varied from 90 minutes to 8 weeks after treatment.

#### Gross pathology

Gross pathology showed well-demarcated ablation zones, discoloration of the ablated tissue and white coagulopathy around the electrodes as early as 90 minutes after treatment[3, 11, 27–29]. The diameter of the ablation zones varied from 1.6 cm to 6.9 cm. The extent of the ablation zone is influenced by several parameters, such as electrode diameter, interelectrode distance and strength of the electric field[37]. No progression in time of the diameter of the ablation zones was reported. In most studies, traversing bile ducts and surrounding vessels remained structurally unharmed[6, 8, 12, 27–29, 31, 32]. In some cases, signs of vasculitis and endothelial necrosis were observed 6 hours post treatment[10, 16, 18]. The same effect was also reported in one study performing histological evaluation 72 hours after treatment[17].

#### Clinical effects

Two studies reported on the systemic effects of IRE ablation on *in vivo* liver tissue[10, 12]. No remarkable signs of hepatitis or biliary obstruction were reported[10]. However, as early as 1 hour after treatment, an increase in both serum alanine aminotransferase (ALT) as well as aspartate aminotransferase (AST) was observed[10]. The blood levels of ALT and AST before treatment were 30 IU/L and 22 IU/L, respectively, and they increased to 44 IU/L and 25 IU/L, respectively one hour after treatment[10]. Six hours after IRE, levels increased to 43 IU/L and 310 IU/L[10]. One study reported an increase in total serum bilirubin in pigs from 3.4 μmol/L to 5.1 μmol/L one hour after IRE, and normalization after six hours[10]. One study reported a peak of bilirubin (4.3 μmol/L) on day 5, which normalized by day 14 (1.7 μmol/L)[12]. No significant change was noted in this study for both ALT and AST up to day 14 after treatment[12].

#### Histopathology

On histopathologic evaluation, eosinophilic cytoplasmatic changes and congestion of sinusoidal spaces were seen immediately after the procedure[36]. These effects persisted at least up to 72 hours after ablation[31]. Furthermore, marked dilatation of the normal sinusoidal spaces without haemorrhagic infiltration was reported[28] as well as oedemic hepatocytes[33] directly following ablation until 90–120 minutes after.

After 90–120 minutes following treatment features characteristic for cell apoptosis were observed, including infiltration of erythrocytes into and between widened sinusoidal spaces [11, 27, 28], as well as condensation of hepatocyte cytoplasm and pyknosis of hepatocyte nuclei [3, 19, 29]. Apoptotic activity was measured using immuno-histochemical staining for caspase-3. This showed wide bands of cleaved caspase-3–positive apoptotic cells at the periphery of the treated ablation zone[11, 27]. Hepatocytes surrounding the electrodes inside the macroscopically visible zone of white coagulation did not show apoptotic activity [3].

Six to seven hours after treatment, hepatocellular degeneration, necrosis, dissociation, rounding of the cells, cytoplasmic hypereosinophilia, nuclear pyknosis, and karyorrhexis were observed[8, 10, 18]. Sinusoidal congestion and haemorrhage, and neutrophilic and lymphocytic infiltration were also seen[16, 32]. In some small and medium-sized blood vessels and bile ducts epithelial necrosis was observed[8, 10, 16, 18, 32].

Evaluation at 24 hours post treatment showed extensive infiltration of inflammatory cells, central coagulative necrosis and a well delineated margin between treated and non-treated tissue[6, 9, 13, 15, 17, 18, 31, 33, 36]. One study reported signs of vasculitis in some vessels, veins and arteries, and acute choledochitis with peridochal oedema[6]. The ablation zone showed increased staining for apoptotic markers BAX or TUNEL compared to surrounding tissue[6, 31].

Forty-eight hours after ablation, areas of vascular congestion and haemorrhagic change were reported with grossly intact hepatic morphology[31]. This was accompanied by neutrophil infiltration and areas of extensive and severe cell death, which was evidenced by pyknotic and hyperchromatic nuclei, eosinophilic cytoplasm[31]. These effects were also seen 72 hours after ablation[31].

Two weeks after ablation, the liver tissue treated with IRE was grossly and histologically normal[12]. The areas of vascular congestion and haemorrhage had resolved[31]. The stroma in the affected areas contains moderate aggregates and scattered inflammatory cells consisting of lymphocytes, plasma cells, and macrophages admixed in some areas with red blood cells[17]. The ablated lesions were almost completely replaced by fibrous scar tissue[17].

Vital tissue such as traversing vessels and bile ducts appeared intact[31].

#### Subgroup analysis

A subspecies analysis was performed for 4 animal types (Pig, Rat, Rabbit and Goat). A delayed inflammatory response in rat (t = 10 hours) was seen compared to pigs (t = 6 hours). No other differences in histological response were identified.

**Fig 2:**
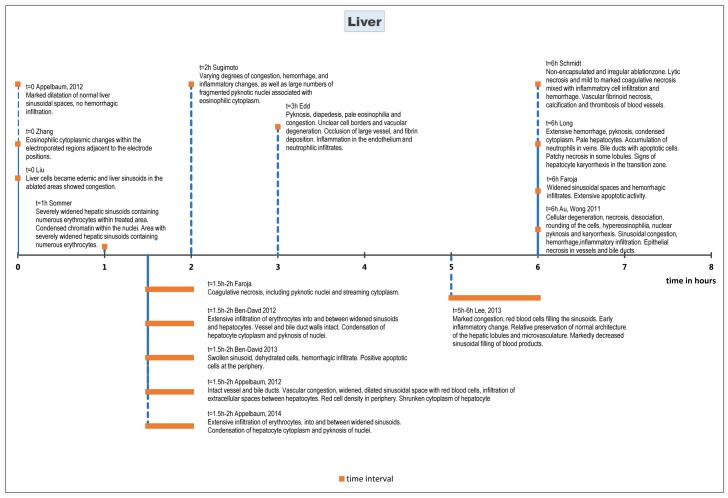
Chronological histological effects of IRE in liver tissue from 0 to 8 hours after treatment.

**Fig 3:**
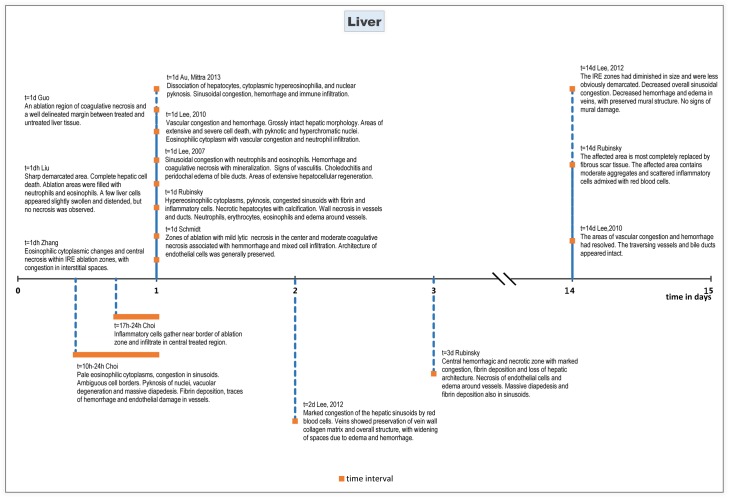
Chronological histological effects of IRE in liver tissue from 10 hours to 15 days after treatment.

Histological effects of IRE on liver tissue are summarized chronologically in Fig 2 (0–8 hours after treatment) and Fig 3 (10 hours– 15 days after treatment).

### Pancreas

Four studies on pancreatic tissue were included, performing IRE in 26 pigs[38–41]. All ablations were performed *in vivo* in healthy pancreatic tissue. All animals survived the treatment. Two animals were excluded from the initial analyses due to improper placement of the electrodes (n = 1), or inability to produce a stable current (n = 1) [38].

Times between IRE and euthanasia varied from 1 hour until 28 days after treatment. The number of pulses varied between 18–90 pulses, electric field strength between 1000 and 3600 V/cm, and pulse length between 50 and 100 μs.

#### Gross pathology

The diameters of the ablation zones varied from 1 cm to 2.3 cm[39–41]. No studies reported on outcomes immediately after treatment. However, as early as 1 hour after treatment, gross pathology showed well-demarcated zones of ablation, with some cases reporting oedema, adhesions and haemorrhage[40]. Oedema and adhesions persisted at least up to 7 days post treatment, whereas haemorrhage was no longer seen at this time of evaluation [39, 40]. None of the included studies reported on the sizes of the ablation zones.

#### Clinical effects

Three studies reported on the systemic effects of IRE on *in vivo* pancreatic tissue[38, 40, 41]. No severe pancreatitis was reported in these studies. Only mild signs of pancreatitis were observed, characterized by a transient increase in white blood cells, amylase and lipase, which normalized by post-ablation day 3[38, 40]. One animal developed a secondary peak in serum amylase, which normalized again after the wound was opened and packed[38]. One study reported elevated ALT levels in pigs, 0–60 U/L above normal levels[38]. This study also reported a transient hypoglycaemia at 1 and 3 hours post-ablation in all animals, which normalized after 24 hours[38]. After 24 hours, serum lipase and amylase levels increased significantly, but normalized after 2 days[40, 41]. An increase in serum LDH and AST was also observed at this time of evaluation[41]. No significant changes in biochemical markers were reported after 14 days post-ablation[41].

#### Histopathology

Microscopy 1 hour after treatment showed oedematous swelling of the interstitium, necrosis of acinar pancreatic tissue and minor foci of interstitial haemorrhage[40]. After two hours, there was histological evidence of haemorrhagic necrosis[39]. Blood vessels and pancreatic ducts appeared more resistant to treatment than acinar pancreatic tissue[40]. Preservation of these structures was reported with no signs of cell death.

Microscopy 24 hours post treatment showed a focally extensive area of complete necrosis with preservation of the lobular architecture, associated with haemorrhage, oedema and a moderate neutrophilic infiltrate[41].

Seven days after treatment, the ablated area was characterized by cellular eosinophilia, beginning fibrosis, and glandular atrophy. The size of the ablated area was similar to the size seen in animals that were euthanized immediately after treatment[40].

After 14 days, the pancreatic tissue appeared either fibrotic (with an inter electrode spacing of 10 mm) or normal (with an inter electrode spacing of 15 mm)[39]. Furthermore, signs of multifocal moderate haemorrhage and haemosiderosis were observed[41]. The parenchyma showed degradation of cells compatible with postmortem autolytic changes[41].

One study performed histological evaluation at 28 days post treatment, and showed diffuse epithelial attenuation and loss in large bile ducts and several small adjacent ducts, surrounded by moderate haemorrhages and fibrosis. The adjacent pancreatic parenchyma showed areas of acinar atrophy and loss, and fibrosis. One specimen, harvested adjacent to a large duct and large vessels, focally showed an extensive area of fibrosis of the interlobular stroma. Some adjacent lobules showed acinar atrophy and loss with fibrosis[41].

#### Subgroup analysis

All pancreas experiments were performed in pigs. Therefore no subspecies analysis of pancreas treated with IRE was performed.

**Fig 4:**
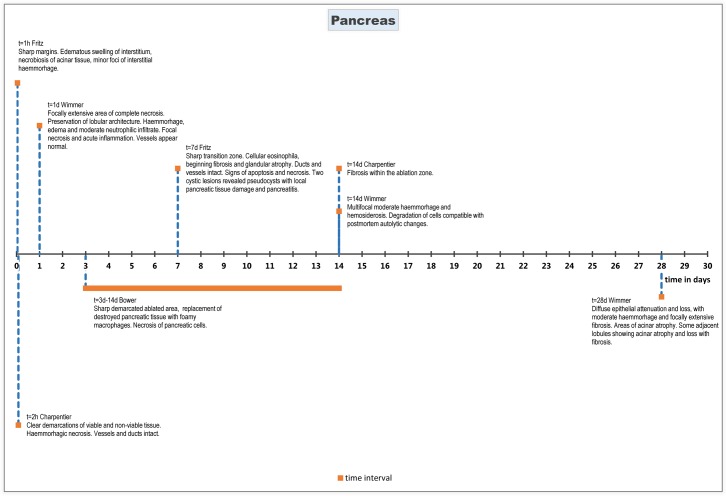
Chronological histological effects of IRE in pancreas tissue from 1 hour to 28 days after treatment.

Histological effects of pancreas tissue treated with IRE are summarized chronologically in Fig 4.

### Blood vessels

Four studies on blood vessels were included using 55 animals[42–45], rats[42, 43, 45] and rabbits[44]. The effects of IRE were observed on solitary large vessels such as the carotid artery[42, 43, 45] or the iliac artery[44]. Between 10 and 90 pulses were delivered, with the electric field varying between 440 and 3800 V/cm. All studies used a pulse length of 100 μs. The ablation of one animal was unsuccessful, because no change in electric conductance was measured and this experiment was not included in the original analysis[43].

#### Gross pathology

Gross pathology was reported in one study, which stated no difference between treated arteries and control arteries. There was no evidence of local bleeding or thrombosis 24 hours after ablation[44].

#### Clinical effects

Both after treatment of carotid as well as iliac arteries, no morbidity (e.g. cerebrovascular events, focal necrosis or bleeding) were reported[42–45].

#### Histopathology

On histological evaluations, no significant changes were reported in vessels treated with IRE up to 24 hours after treatment[42]. Histological changes were reported 7 days after treatment, reporting a lower number of vascular smooth muscle cells in the tunica media of treated arteries compared to control arteries[42, 44]. The endothelial cells and the internal lamina morphology were preserved. Compared to control arteries, the endothelial layer of the IRE treated arteries was thinner and more condensed. Location of elastic fibers and morphology were not different between the IRE arteries and the control arteries[42, 44, 45]. Most of the inflammatory response was perivascular[44]. There was no evidence of thrombosis, aneurysm formation or rupture of the arteries. At the time of evaluation, apoptotic cells were seen in the most internal layer of the tunica media. This layer also included some clusters of vascular smooth muscle cells. One study reported mild fibrosis in the perivascular area with dominance of collagen fibers in the tunica media[45].

There was no histology reported between 7 days and 28 days post treatment. Histology at 28 days after treatment showed a reduction of vascular smooth muscle cells in the tunica media compared to the control artery[42, 43]. There was no evidence of thrombus formation and no change in the diameter of the artery. The endothelial cells and the internal lamina morphology were preserved. Compared to the control artery, the endothelial layer of the IRE treated arteries was thinner and more condensed[42].

One study reported histological evaluation 35 days post treatment, which showed circumferential damage in nearly all sections of the treated artery. Damage was characterized by persistent loss of vascular smooth muscle cells, with the tunica media replaced by fibroblasts and fibrotic, collagenous tissue. In most cases, the elastic lamina remained intact. Occasional mural inflammation was noted; this included primarily mononuclear cells, and also polymorphonuclear cells (neutrophils). Most of the chronic inflammatory reaction was perivascular, but some of the segments showed inflammatory cells through the wall. Some of the treated arteries showed asymmetric, fibrous non-cellular neo-intima covering the intimal surface[44].

#### Subgroup analysis

A subspecies analysis was performed for two animal types (rabbit, rat). Fibrosis was seen at a later time of evaluation in rabbits (t = 35 days) compared to rats (t = 7 days). All other observations are similar between animal types.

**Fig 5:**
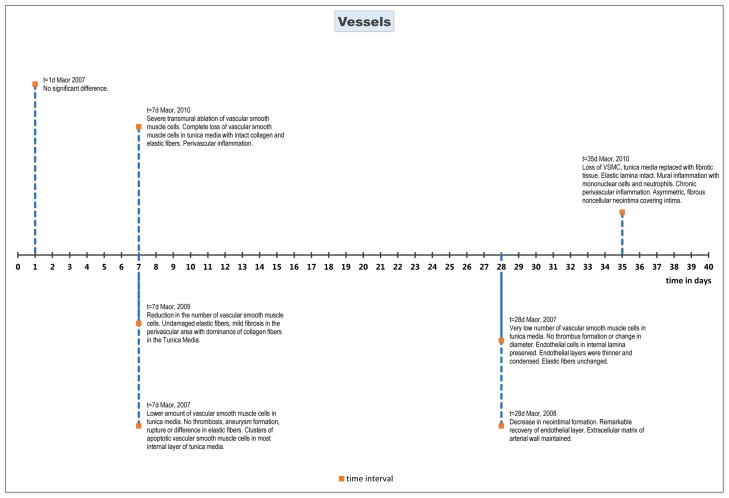
Chronological histological effects of IRE on blood vessels from 1 to 35 days after treatment.

Histological effects are summarized chronologically in Fig 5.

### Nerves

Four studies on nerves were included using 84 animals[38, 46–48]. Animals used were either pigs[46–48] or rats[38]. All animals survived the treatment and all ablations were successfully performed. The electric field varied between 1500 and 3800 V/cm, number of pulses from 10 to 90 and pulse length from 70 to 100 μs.

#### Gross pathology

Gross pathology of treated tissue showed well-demarcated, focal lesions[46–48]. One study reported lesions that were pale tan, firm, and contained numerous small (1 mm in diameter) hard white foci of calcification[39].

#### Clinical effects

Li et al. and Wong et al. reported no differences in nerve function immediately after treatment between controls and treated nerves[48, 49].

Most animals were able to put weight on as well as walk on the treated limb within 1–4 days (n = 20), but some animals were incapable of bearing any weight on the treated limb at this time after ablation (n = 2) [46–48].

The sciatic functional index (SFI) measured by Li and colleagues 3 days after ablation showed a significant decrease compared with control nerves. The functional index of treated sciatic nerves showed an increase from 3 weeks after treatment, but remained significantly decreased up to 5 weeks after ablation. By 7 weeks, no significant decrease in SFI was measurable between IRE treated nerves and controls[49].

#### Histopathology

In one study, all nerves showed perineural edema and infiltration by a few eosinophils and neutrophils 24 hours after ablation. In five out of six nerves, axonal swelling was observed[46].

Three days after ablation, myelin degeneration and perineural infiltration by a small number of neutrophils and eosinophils was observed. Furthermore, decreased protein expression was observed around many axons when compared to the control nerve, indicating Schwann cell loss[38, 46].

On day 6 after IRE ablation, there was an increased cellularity within the affected fascicles, characterized by cells with ovoid elongated nuclei and scant cytoplasm, suggesting proliferation of both Schwann cells and fibroblasts[46]. One nerve showed fragmentation of axons and presence of phagocytes within ellipsoids[46]. Multifocal, dense clusters of cells were seen. Some of these cell clusters demonstrated S100 expression and Büngner band formation, indicating proliferation of Schwann cells[46].

The same changes were observed 14 days after ablation, with now more extensive and advanced fragmentation of axons, infiltration by phagocytes, and endoneural hypercellularity[46]. A mild increase in perineural collagen was observed in one study[47].

Twenty-one days after ablation, no histological changes in muscle, perimuscular soft tissue, and neurovascular bundles were reported[48].

Finally, one study performed histological evaluation two months post treatment, which showed diffuse hypercellularity within the affected fascicles, with almost all cells expressing S100. Large numbers of small caliber axons expressing neurofilaments were closely associated with hyperplastic Schwann cells, consistent with axonal regeneration. Ellipsoids, resulting from fragmentation of myelin sheets and axons were present in minimal numbers. There was a small increase in the amount of endoneural and perineural collagen[47].

#### Subgroup analysis

A subspecies analysis was performed for 2 animal types (pig, rat). The initiation of a histological response, as well as the onset of regeneration was significantly earlier in pigs (first response t = 24h and regeneration t = 6d) compared to rat (first response t = 3d and regeneration t = 3wk). No inflammatory response was seen in rat nerves treated with IRE.

**Fig 6:**
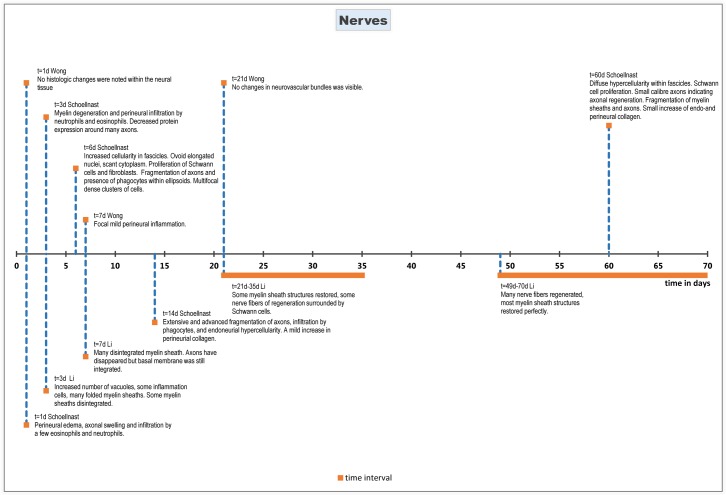
Chronological histological effects of IRE on nerves 1 to 70 days after treatment.

Histological effects are summarized chronologically in Fig 6.

## Discussion

This is the first systematic review to summarize the time-dependent impact of irreversible electroporation. The ablation zone of tissue treated with IRE shows a dynamic process, with changes taking place over 70 days after treatment and varying per tissue type.

Firstly, it is interesting to note that all treated tissue types show a rather similar progression of the ablation zone, but that the time interval differs between tissue types. Whereas liver[11, 27] and pancreas[40] showed a relatively quick histopathological response within hours after IRE, this may take up to 24 hours in nerves[46] and even 7 days in blood vessels[42, 44]. This variety in onset of a histopathological response in different tissue types after IRE may be explained by varying tissue properties. Whereas nerves exist mainly out of post-mitotic cells, naturally these structures should prohibit cell death through apoptosis to remain vital, given the fact that regeneration of these structures is limited[50]. Natural defence mechanisms may therefore prohibit apoptosis from occurring earlier within the process of tissue damage. For nerves, a contributing factor to inhibition of apoptosis could be the Neuronal Apoptosis Inhibitory Protein(NAIP)[51]. This protein is a natural inhibitor of caspase-induced apoptosis, which is associated with programmed cell death after IRE. Likewise, staining of the ablation zone for apoptotic activity with caspase-3 shows increased uptake within these treated regions on histological evaluation[11, 27]. Therefore it can be assumed that inhibition of caspase by NAIP slows down the process of cell death, explaining the relatively late onset of a histological response in nerves treated with IRE. Likewise, an inhibitory factor of apoptosis in endothelial cells of blood vessels could be serum albumin[52]. As with NAIP, it has been demonstrated in previous experiments that albumin plays a role in the inhibition of apoptosis in blood vessels[52]. Under normal circumstances, it is believed that albumin inhibits apoptosis from occurring in blood vessels, to allow for survival of perfused vessels. Likewise, non-functional vessels are effectively removed through apoptosis[52]. However, the role of NAIP and albumin as inhibitors of apoptosis through IRE in blood vessels and nerves has not been defined yet.

Despite the extensive pathological response, the ablation may only lead to a mild pancreatitis and hepatitis, but without clinical signs of pain or discomfort[9, 10, 12, 38, 40, 41]. This may be a favourable factor for clinical application of IRE in pancreas and liver. Previously reported clinical studies comparing thermal ablation (RFA) with IRE show a reduced morbidity and similar pain levels in favour of IRE[53].

Although IRE is thought to be a non-thermal technique[3], IRE-treated lesions often show a centre of white coagulation surrounding the electrodes[3, 27–29, 35], histologically characterized by streamlined cytoplasm and pyknotic nuclei[28]. This is suggestive for thermal necrosis caused by an increase in temperature during treatment[3, 27–29, 35]. In general, these effects are seen while using electric field strengths above 1200 V/cm. Histologically, less apoptotic activity (and more necrosis) is seen within these zones while using an immunohistochemical apoptosis marker such as caspase-3 [3]. Therefore, the non-thermal mechanism remains under discussion[3], and careful treatment planning ought to be applied in order to spare structures vulnerable to thermal damage.

In order to allow ablations to be performed in rat liver, Choi et al used a custom designed microfabricated electroporator (MFE) [13]. The electric field strength distribution was similar to clinical two-needle electroporators. The size of the ablation zones created with a MFE was significantly smaller, but showed faster regeneration in time[13]. Up to 10 hours after treatment, no significant difference in extent of the ablation zone, geometry or apoptotic activity could be observed compared to clinical electroporators. Yet, after 17 hours, a rapid decrease in apoptotic activity as identified by the TUNEL-assay was observed compared to clinical electroporators. This could be related to the faster speed of wound healing due to the reduced size of the ablation zones[13].

Some studies reporting contradicting results of similar experiments. Firstly, Maor and colleagues performed IRE on solitary vessels and reported an inflammatory reaction after treatment[44], while in a second study, no inflammatory response was observed[42, 43]. The main differences in these experiments concerned animals used (rabbits vs rats) and treatment settings.[42, 44, 45]. The variation in effect could also be explained by the fact that a higher voltage was used in one experiment causing more extensive necrosis, and therefore, causing less inflammatory reaction in the treated tissue.

Secondly, according to the results of this review, non-pathological liver tissue treated with IRE is capable of regenerating within fourteen days after treatment. On histopathological examination, the liver samples were grossly and histologically normal at this time point of evaluation[12]. Therefore it could be expected that this accounts for human liver tissue treated with IRE as well, and tumorous tissue could even be replaced with fibrotic tissue or resolve completely after this time[31, 34]. However, Cheng and colleagues performed a resection following IRE with a median of 10 months (range 3–17 months) after ablation on liver in patients diagnosed with hepatocellular carcinoma[54]. Tumors treated with IRE showed sharp demarcated zones of confluent necrosis at this time of evaluation, rather than fibrosis or pathologically normal tissue. This might be explained by a different histopathological response of healthy and pathological tissue[55]. In contrast to healthy tissue, pathological tissue may be less capable of regeneration, explaining the longer duration of necrosis reported by Cheng and colleagues[54].

In addition, as described in the animals experiments included in this review, ablation zones in liver tissue showed well-demarcated lesions as early as 90–120 minutes after IRE [11, 27, 28]. Likewise, Scheffer and colleagues performed IRE on colorectal liver metastases in humans (COLDFIRE-1 study), with a median resection time of 84 minutes (range 51–153 minutes) after ablation[56]. The reason for excluding the results of our COLDFIRE-1 study was due to the pathological nature of the treated tissue. The pathological features of colorectal liver metastases are likely aberrant from healthy liver tissue.

The tumor-free margin surrounding the ablation zone showed histological features corresponding to those of previously reported animal experiments. However, the treated tumor region was highly heterogeneous, characterized by unevenly distributed intensity of apoptotic activity. TTC staining of the ablation zone proved a-vitality in the treated regions, but microscopically this was harder to objectify. Several factors could have contributed to these results. One theory for the uneven distribution of apoptotic activity could be that pre-existing tumor necrosis may have prohibited apoptosis from occurring. Another theory could be that the time between IRE and resection could have been too short to allow cell death to be completed[56]. As shown in this review, the first characteristics of apoptosis can be seen 90–120 minutes after IRE[11, 27, 28], which is comparable to the time of evaluation as performed by Scheffer and colleagues (median resection time of 84 minutes after IRE) [56]. However, apoptosis progresses up to 24 hours after ablation[6, 31], therefore it can be assumed that the process of cell death had not been completed yet during the time of evaluation.

A contributing factor to tumor death may also be the secondary effects of ischemia and hypoxia caused by necrosis of the surrounding tissue, which was previously reported by Guo and colleagues in a rat experiment treating hepatocellular carcinoma[57]. However, as reported in this review, this process may take up to 24 hours in order to be completed. Therefore the effect of IRE on tumor treatment in this experiment may have been underestimated.

This review gives an overview of the time-dependent impact of IRE on pancreas, liver, blood vessels and nerves. One limitation of this systematic review is the limited availability of data on the histological response and progression in time of pathologic tissue treated with IRE. Therefore, it cannot automatically be stated that the effects observed in the included studies are representative for the effects when IRE is used in clinical practice in the treatment of malignancies. Another limitation is that none of the studies reported on the progression of the ablation zone in time, but all had a fixed time of histological evaluation, while the effect of IRE is clearly a dynamic process. Therefore, data are missing at certain times of evaluation, creating gaps in time. Moreover, the histological changes are highly influenced by the settings used, which explains the different outcomes observed at the same time of evaluation. Also, most studies did not include a control group which hampers comparison.

Finally, further research is needed to demonstrate the effects of IRE as a dynamic process, and to obtain the optimal settings for treatment. This could involve studies in varying tissues, on varying time points after IRE to validate the findings of previously performed animal experiments, comparable animal experiments in pathological tissue (e.g. a hepatocellular carcinoma or pancreatic carcinoma model) and subsequently the use in clinical practice.

Moreover, due to the relatively small numbers of studies included for pancreas (n = 4), blood vessels (n = 4) and nerves (n = 4) and the absence of a statistical test for qualitative results, the significance, and thus the risk of bias, could not accurately be determined. Likewise, this also applies for the interspecies variety within animals treated with IRE included in this review, of which the influence on histopathological outcomes after IRE could not accurately be determined. Similar experiments could be performed in varying animal species, to allow for determination of the influence of varying animal types on pathological outcomes.

## Conclusion

Tissues treated with IRE show a distinct dynamic process over time. A predictable pattern can be observed, starting with infiltration of erythrocytes, followed by inflammatory infiltration and progressive cell apoptotic activity, but timing of effects varies with different tissue types.

## Supporting Information

S1 Appendix Search strategy. (PDF)

S2 Appendix List of definitions. (PDF)

S1 Table Inclusion and exclusion criteria for study eligibility. (PDF)

S2 Table Assessment of methodological quality of included studies. (PDF)

S3 Table Subgroup analysis of influence of animal species on tissue response in time after IRE on liver, pancreas, blood vessels and nerves. (PDF)
