# Correction: Time-Dependent Impact of Irreversible Electroporation on Pancreas, Liver, Blood Vessels and Nerves: A Systematic Review of Experimental Studies

**DOI:** 10.1371/journal.pone.0174018

**Published:** 2017-03-10

**Authors:** J. A. Vogel, E. van Veldhuisen, P. Agnass, J. Crezee, F. Dijk, J. Verheij, T. M. van Gulik, M. R. Meijerink, L. G. Vroomen, K. P. van Lienden, M. G. Besselink

There are errors in the Results and Conclusions sections of the Abstract.

The last sentence of the Results section of the Abstract should read: In nerves, the median time until demonstrable histological changes was 24 hours.

The last sentence of the Conclusions section of the Abstract should read: Whereas pancreas and liver showed the first damages after 1–2 hours, this took 24 hours in nerves and 7 days in blood vessels.

## References

[1] Vogel JA, van Veldhuisen E, Agnass P, Crezee J, Dijk F, Verheij J, et al (2016) Time-Dependent Impact of Irreversible Electroporation on Pancreas, Liver, Blood Vessels and Nerves: A Systematic Review of Experimental Studies. PLoS ONE 11(11): e0166987 doi:10.1371/journal.pone.0166987 27870918 10.1371/journal.pone.0166987 PMC5117758

