# Supplementary material for: Time-Dependent Impact of Irreversible Electroporation on Pancreas, Liver, Blood Vessels and Nerves: A Systematic Review of Experimental Studies
Source: PLoS One. 2016 Nov 21;11(11):e0166987. doi: 10.1371/journal.pone.0166987 (PMC5117758; doi:10.1371/journal.pone.0166987)
Supplement: S1 Appendix — (PDF) [file pone.0166987.s001.pdf]

S1 Appendix: Search strategy

Search strategy PubMed

(irreversible electroporation[tiab] OR N-TIRE[tiab] OR Nanoknife[tiab] OR IRE[tiab]) AND (pathology[tiab] OR pathologic*[tiab] OR microscop*[tiab] OR tissue[tiab] OR cell[tiab] OR necrosis[tiab] OR apoptosis[tiab] OR size[tiab])

Search strategy Embase

(exp. irreversible electroporation or N-TIRE.mp [mp=title, abstract, heading word, drug trade name, original title, device manufacturer, drug manufacturer, device trade name, keyword] or irreversible electroporation.mp. [mp=title, abstract, heading word, drug trade name, original title, device manufacturer, drug manufacturer, device trade name, keyword] or IRE.mp. [mp=title, abstract, heading word, drug trade name, original title, device manufacturer, drug manufacturer, device trade name, keyword]) and (pathology.mp. [mp=title, abstract, heading word, drug trade name, original title, device manufacturer, drug manufacturer, device trade name, keyword] or pathologic.mp. [mp=title, abstract, heading word, drug trade name, original title, device manufacturer, drug manufacturer, device trade name, keyword] or microscop*.mp. [mp=title, abstract, heading word, drug trade name, original title, device manufacturer, drug manufacturer, device trade name, keyword] or tissue.mp. [mp=title, abstract, heading word, drug trade name, original title, device manufacturer, drug manufacturer, device trade name, keyword] or cell.mp. [mp=title, abstract, heading word, drug trade name, original title, device manufacturer, drug manufacturer, device trade name, keyword] or necrosis.mp. [mp=title, abstract, heading word, drug trade name, original title, device manufacturer, drug manufacturer, device trade name, keyword] or apoptosis.mp. [mp=title, abstract, heading word, drug trade name, original title, device manufacturer, drug manufacturer, device trade name, keyword] or size.mp. [mp=title, abstract, heading word, drug trade name, original title, device manufacturer, drug manufacturer, device trade name, keyword])

Search strategy Cochrane

Irreversible electroporation (title, abstract, keywords) OR N-TIRE (all text)
