# Supplementary material for: Time-Dependent Impact of Irreversible Electroporation on Pancreas, Liver, Blood Vessels and Nerves: A Systematic Review of Experimental Studies
Source: PLoS One. 2016 Nov 21;11(11):e0166987. doi: 10.1371/journal.pone.0166987 (PMC5117758; doi:10.1371/journal.pone.0166987)
Supplement: S2 Appendix — (PDF) [file pone.0166987.s002.pdf]

S2 Appendix: List of definitions

Apoptosis – *programmed cell death*

Büngner band – *column of collapsed Schwann cell*

Condensation (of nuclei or cytoplasm) – *becoming more dense*

Congestion – *excessive fluid*

Diapedesis – *movement or passage of blood cells.*

Ellipsoid – *digestion chamber containing axonal granules*

Haemosiderosis – *accumulation of haemosiderin (iron-storage complex)*

Karyorrhexis – *rupture of cell nucleus*

Necrosis – *cell death by enzymatic degradation*

Pyknosis – *irreversible condensation of chromatin in the nucleus*

Sinusoidal space – *interstitial space between cells*
