# Supplementary material for: Time-Dependent Impact of Irreversible Electroporation on Pancreas, Liver, Blood Vessels and Nerves: A Systematic Review of Experimental Studies
Source: PLoS One. 2016 Nov 21;11(11):e0166987. doi: 10.1371/journal.pone.0166987 (PMC5117758; doi:10.1371/journal.pone.0166987)
Supplement: S1 Table — (PDF) [file pone.0166987.s003.pdf]

S1 Table: Inclusion and exclusion criteria for study eligibility.

**Inclusion criteria**
- Treatment with irreversible electroporation
- Pathology evaluation
- Experimental studies
- Liver, pancreas, blood vessels or nerves
- Time indication of histological evaluation
- Original articles

**Exclusion criteria**
- Purely thermal therapies
- In silico/mathematical models
- Pathological tissue
- IRE in non-animal tissue
