# Supplementary material for: Time-Dependent Impact of Irreversible Electroporation on Pancreas, Liver, Blood Vessels and Nerves: A Systematic Review of Experimental Studies
Source: PLoS One. 2016 Nov 21;11(11):e0166987. doi: 10.1371/journal.pone.0166987 (PMC5117758; doi:10.1371/journal.pone.0166987)
Supplement: S2 Table — (PDF) [file pone.0166987.s004.pdf]

S2 Table: Assessment of methodological quality of included studies.

	Randomisation	Allocation concealed	Identical conditions	Blinded researchers	Complete data	Exposure characterization	All outcomes reported	Threats to internal validity	Outcome assessment
	n/a	n/a	PL	n/a	DL	PL	PL	PL	PL
Appelbaum[28]	n/a	n/a	PL	n/a	PL	PH	PH	PL	PL
Au[8]	n/a	n/a	PL	n/a	PL	PL	PL	PH	PL
Au[9]	n/a	n/a	PL	n/a	PL	PL	PL	PL	PL
Au[10]	n/a	n/a	PL	n/a	PL	PL	PL	PL	PL
Ben-David[11]	n/a	n/a	PL	n/a	DL	PL	PL	PL	PL
Ben-David[29]	n/a	n/a	PL	n/a	PL	PL	PL	PL	PL
Charpentier[12]	n/a	n/a	PL	n/a	PL	PH	PH	PL	PH
Choi[13]	n/a	n/a	PL	n/a	PL	PL	PL	PL	PL
Edd[14]	n/a	n/a	PL	n/a	DL	PL	PL	PL	PL
Faroja[3]	n/a	n/a	PL	n/a	DL	PL	PL	PL	PL
Golberg[30]	n/a	n/a	PL	n/a	PH	PH	PH	PL	PL
Guo[15]	n/a	n/a	PL	n/a	DL	PL	PL	PL	PL
Lee[31]	n/a	n/a	PL	n/a	DL	PL	PL	PL	PL
Lee[6]	n/a	n/a	PL	n/a	PH	PL	PH	PL	PL
Lee[32]	n/a	n/a	PL	n/a	PL	PL	PL	PL	PL
Lee[33]	n/a	n/a	PL	n/a	PL	PL	PL	PL	PL
Liu[16]	n/a	n/a	PL	n/a	PL	PL	PL	PL	PL
Long[34]	n/a	n/a	PL	n/a	PL	PH	PL	PL	PH
Rubinsky[17]	n/a	n/a	PL	n/a	DL	DL	DL	PL	PL

Schmidt[18]	n/a	n/a	PL	n/a	PH	PL	PH	PL	PL
Sommer[35]	n/a	n/a	PL	n/a	PL	PL	PL	PL	PL
Sugimoto[19]	n/a	n/a	PL	n/a	DL	PL	PL	PL	PL
Zhang[36]	n/a	n/a	PL	n/a	PL	PH	PH	PL	PH
Bower[38]	n/a	n/a	PL	n/a	PH	PH	PH	PL	PH
Charpentier[39]	n/a	n/a	PL	n/a	DL	PL	PL	DL	PL
Fritz[40]	n/a	n/a	PL	n/a	PL	PL	PL	PL	PL
Wimmer[41]	n/a	n/a	PL	n/a	DL	PH	PH	PH	PH
Maor[42]	n/a	n/a	PL	n/a	PL	PL	PL	PL	PL
Maor[43]	n/a	n/a	PL	n/a	PL	PL	PL	PL	PL
Maor[44]	n/a	n/a	PL	n/a	PL	PL	PL	PL	PL
Maor[45]	n/a	n/a	PL	n/a	PL	PH	PL	PL	PL
Li[38]	n/a	n/a	PL	n/a	DL	PL	PL	PL	PL
Schoellnast[46]	n/a	n/a	PL	n/a	DL	PL	PL	PL	PL
Schoellnast[47]	n/a	n/a	PL	n/a	DL	PL	PL	PL	PL
Wong[48]	n/a	n/a	PL	n/a	DL	PL	PL	PL	PL

Assessment of methodology according to the Office of Health Assessment and Translation criteria for human and animal studies[26].

DL: definitely low risk; PL: probably low risk; PH: Probably high risk; DH: definitely high risk; n/a: not applicable.
