# Supplementary material for: Time-Dependent Impact of Irreversible Electroporation on Pancreas, Liver, Blood Vessels and Nerves: A Systematic Review of Experimental Studies
Source: PLoS One. 2016 Nov 21;11(11):e0166987. doi: 10.1371/journal.pone.0166987 (PMC5117758; doi:10.1371/journal.pone.0166987)
Supplement: S3 Table — (PDF) [file pone.0166987.s005.pdf]

S3 Table: Subgroup analysis of influence of animal species on tissue response in time after IRE on liver, pancreas, blood vessels and nerves.

Liver	First response	Cell death	Inflammation	Fibrosis
Pig	t=0	t=90-120m	t=6h	t=24h
Rat	t=0	t=3h	t=10h	N/A (N/P 24h)
Rabbit	N/A	N/A	N/A	N/A
Goat	t=0	N/A	N/A	N/A (N/P 24h)

Pancreas	First response	Cell death	Inflammation	Fibrosis
Pig	t=0	t=2h	N/A	t=7d

Blood vessels	First response	Cell death	Inflammation	Fibrosis
Rabbit	N/A	t=7d	t=7d	t=35d
Rat	N/A	t=7d	t=7d	t=7d

Nerves	First response	Cell death	Inflammation	Regeneration
Pig	t=24h	t=3d	t=24h	t=6d
Rat	t=3d	t=3d	N/A	t=3w

N/A = not derivable from extracted data, N/P = not present at given sample time, t = time, m= minutes, h = hours, d = days, w = weeks.
